# The mutagenic and antimutagenic activity of *Sutherlandia frutescens* extracts and marker compounds

**DOI:** 10.1186/s12906-018-2159-z

**Published:** 2018-03-15

**Authors:** Siyabulela S. B. N. Ntuli, Wentzel C. A. Gelderblom, David R. Katerere

**Affiliations:** 1 Department of Conservation and Marine Sciences, Cape Peninsula University of Technology, P.O. Box 654, Cape Town, 8000 South Africa; 2 Institute of Biomedical and Microbial Biotechnology, Cape Peninsula University of Technology, P O Box 1906, Bellville, 7535 South Africa; 3 Department of Pharmaceutical Sciences, Tshwane University of Technology, Staatsartillerie Road, Pretoria West, Pretoria, 0183 South Africa

**Keywords:** Fabaceae, *Sutherlandia frutescens*, Total polyphenols, Mutagenic activity, Antimutagenic activity, Antioxidant activity, Promutagenicity

## Abstract

**Background:**

*Sutherlandia frutescens* (L.) R. Br is endemic to Southern Africa where it has been traditionally used for cancer and diabetes. In recent times it has been marketed for its reputed (but not proven) anticancer, antidiabetic and anti-HIV properties. Little is known about the mutagenic and antimutagenic potential of extracts and common marker compounds of *Sutherlandia frutescens*. Therefore this study aimed to investigate the putative efficacy and possible long-term adverse effects of using this herb.

**Methods:**

Ethylacetate (EA) and 50% Methanol (MeOH) extracts were screened for mutagenic and antimutagenic activity using the Ames assay utilising TA97a, TA98, TA100 and TA102 in the presence and absence of metabolic activation. Four compounds, L-arginine, L-canavanine, GABA and D-pinitol known to occur in sutherlandia were also included. The total polyphenolic content of the both extracts was determined using the Folin-Ciocalteau method and FRAP and ABTS were used to determine the anti-oxidant potential of the extracts.

**Results:**

The extracts and the standards did not show any cytotoxicity except in TA97a. The EA extract exhibited antimutagenicity against all the bacterial strains at all concentrations tested. The MeOH extract showed both pro-mutagenic and antimutagenic activities with 2-acetamidofluorene and aflatoxin B1 in the presence of metabolic activation of TA98 and TA100, respectively. All compounds, except L-canavanine exhibited antimutagenic activity against all strains. L-canavanine, on the other hand showed co-mutagenicity with 9-aminoacridine on TA97a, at all test concentrations. The extracts and pure compounds exhibited their antimutagenic activity in a dose response manner. L-arginine and GABA showed an some antimutagenic response. EA extract had three times the total phenolic content (12.56 μg GE / mg) observed in the MeOH extract. There was correlation between total phenolic content, antioxidant potential and antimutagenicity.

**Conclusion:**

Both extracts exhibited a protective effect, with the EA extract exhibiting greater potency. L-canavanine acted as a co-mutagen in a dose response manner without metabolic activation. It is suggested that the EA extract be priotized for future development work as it showed a better risk profile and activity.

## Background

Southern Africa boasts a rich floral diversity which is utilized in traditional herbal medicine. It is estimated that 27–30 million South Africans rely on traditional medicine for their primary health care needs [1, 2]. There are about 30,000 species of higher plants indigenous to Southern Africa of which only about 10% are used as traditional medicines [3]. Due to the long history of cultural use, medicinal plant species are often assumed to be safe, with the public perceiving that they are ‘natural’ [4, 5]. This perception is largely unfounded.

*Sutherlandia frutescens* (eq. *Lessertia frutescens*) (L.) R. Br. (Fabaceae, formerly Leguminosae, sub-family Papilionoideae) also known locally as cancer bush, kankerbos (Afrikaans) and unwele (Zulu), is widely marketed in South Africa and elsewhere as sutherlandia tablets or as a leaf decoction or infusion. It is reputed, by various ethnic groups including the Zulus, Khoi-San and Xhosas, to treat a number of different diseases including diabetes mellitus, cancer, stomach complaints, topical wounds, gonorrhea and syphilis [6, 7], stress, depression and inflammation/arthritis [8, 9]. In more recent times it has also been used in the management of HIV/AIDS [10–13]. Chinkwo [14] showed that it induced apoptosis in cultured carcinoma cells while [15] showed that it possessed antibacterial and antioxidant activity. The biological activity of *S. frutescens* has been attributed to the presence of various compounds including L-canavanine, D-pinitol, gamma (γ) aminobutyric acid (GABA) [16] and cycloartane glycosides such as sutherlandioside A, B, C and D [13, 17]. In spite of its widespread use as a herbal medicine, there are few preclinical studies done to support the various medicinal claims [18]. Furthermore, there is little known about its potential for cellular toxicity.

**Fig. 1:**
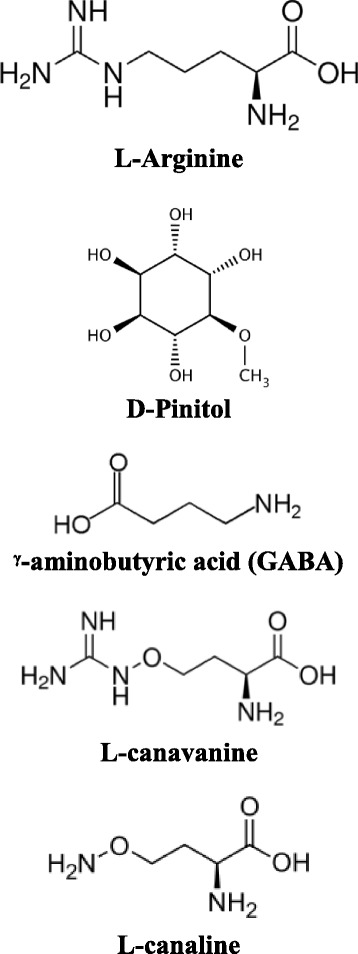
Structures of the pure compounds from *S. frutescens*

The present study investigated the mutagenic and antimutagenic potential of sutherlandia and its known constituents i.e. L-arginine, asparagine, GABA, L-canavanine, and pinitol (Fig. 1). L-arginine is an essential amino acid in protein synthesis and has been reported to exhibit anti-diabetic activity [19]. It has been reported to attenuate the anti-proliferative activity of L-canavanine, for which it is a structural analogue [20].

L-canavanine is a non-proteinogenic amino acid present in most legumes. It is a potentially toxic isomer and antimetabolite of L-arginine [21]. Ames et al. [22] reported that plants utilize L-canavanine to minimize and/or prevent predation. L-canavanine may be potentially beneficial as it has been shown to possess anticancer activity, particularly against pancreatic cancer [23], antiviral and antiretroviral actions [7] and to selectively inhibit inducible nitric oxide synthase (iNOS), which action is of therapeutic importance in septic shock and chronic inflammation [24, 25].

GABA is the major inhibitory neurotransmitter in the CNS. Its low levels in the cerebrospinal fluid have been linked to epilepsy, schizophrenia and Alzheimer’s disease [26] and bipolar and mood disorders [27, 28]. It has purported anti-aging and antioxidant properties [29] and inhibits tumor cell migration [30]. The sugar D-pinitol (a 3-methoxy analogue of D-chiroinositol) has been patented as an anti-diabetic and anti-cachexial agent [31, 32].

### Mutagenicity of south African plants and sutherlandia

There has been considerable interest in testing for mutagenicity and antimutagenicity [33] of traditional herbal medicines [34]. Mutagenicity of more than 50 South African plant species used in traditional medicine was reported by [4] while [35] reported on the mutagenic and antimutagenic of extracts of various important plant species, including *S. frutescens*. In view of the widespread use and therapeutic and/or toxic potential of sutherlandia, the present study investigated its cytotoxic, mutagenic and antimutagenic potential. This was done to understand how sutherlandia extracts and pure compounds exert their putative chemopreventive properties and if their use may be deleterious at cellular level. In addition, the polyphenolic content was assayed as this is so often linked to antioxidant activity which in turn is linked to antimutagenic activity. These polyphenolic compounds are compounds found in nature and abundant in plants and have one or more hydroxyl groups attached directly to an aromatic ring or a benzyl ring [36].

## Methods

### Ames assays

#### Reagents and media

Whole plant material of *Sutherlandia frutescens* was purchased from a commercial phytomedicine supplier Parceval Pharmaceuticals (Pty) Ltd, Wellington, R.S.A. The batch number was 1,009,054 and voucher specimen (dated 20.11.2006) is deposited in the company’s herbarium in Wellington, Cape Town. The four pure compounds (Fig. 1), L-arginine, L-canavanine, D-pinitol and GABA were purchased from Merck Chemicals (Pty) Ltd (Darmstadt, Germany). All other chemicals and reagents were of analytical grade.

The mutagenic compounds i.e. 2-acetamidofluorene (2-AAF), aflatoxin B_1_ (AFB_1_), 9-aminoacridine (9-AA), as well as nicotine adenine dinucleotide phosphate (NADP) and glucose-6-phosphate (G-6-P) were obtained from Sigma-Aldrich, South Africa and cumoyl hydroperoxide (CHP) was purchased from Merck Chemicals (Pty) Ltd, Darmstadt, Germany. Bacto agar and Nutrient Broth No. 2 were purchased from Difco Laboratories (Detroit, USA), Oxoid (Hampshire, UK), D-Biotin and L-(−)-Histidine were purchased from ICN Biomedicals Inc. (Ohio, USA) and Merck Chemicals (Pty) Ltd, (Darmstadt, Germany) respectively. The aroclor-1254 was obtained from Monsanto, St Louis, USA. The *Salmonella typhimurium* strains TA97a, TA98, TA100 and TA102 were obtained from Prof B. N. Ames, Berkeley University, CA, USA.

### Preparation of crude extracts

Sutherlandia (100 g) was extracted with 3 × 600 ml of ethylacetate (EA) or 50% methanol (MET) in a Polytron PT 3100 blender (Kinematica, Switzerland) between 8000 and 8500 rpm for about 25 min. The pooled extracts were then centrifuged at 3000 rpm for 10 min in a Sorvall® Refrigerated Centrifuge (Sorvall® Instruments, Newtown, USA), filtered through a Whatman No. 1 filter paper (Whatman International Ltd, Maidstone, England) and then dried on a rotary evaporator (Buchii, Switzerland), at about 50 °C. All crude extracts were kept desiccated at 4 °C. In the case of aqueous methanol, freeze-drying was done after removing the methanol.

### Preparation of working solutions

Stock solutions of the crude extracts and pure compounds were prepared and subsequent dilutions made in 100% DMSO before conducting all the assays. In all cases, preliminary sub-toxic dose finding was done. The EA extract was diluted to 5, 10 and 20% (*w*/w) whilst the 50% MeOH solution extract was diluted to 10, 25 and 50% (w/w). The pure compounds were each dissolved and diluted to 1, 2.5 and 5% (w/w) concentrations resulting in the following molar concentrations: L-arginine (0.05, 0.14 and 0.28 M); L-canavanine (0.05, 0.13 and 0.26 M); D-pinitol (0.05, 0.13 and 0.26 M); and GABA (0.10, 0.24 and 0.49 M). In all cases the concentrations were determined by toxicity exhibited in the dose finding assays.

### *Salmonella* mutagenicity assay

#### Bacterial strains and mutagens

The strains selected for the study were TA97a (to induce deletion mutations), TA98 (for frame-shift mutations), TA100 (for base-pair substitution mutations) and TA102 (for mutation due to oxidative stress caused by free-radicals). Diagnostic mutagens were 9-aminoacridine (9-AA), 2-acetamidofluorene (2-AAF), aflatoxin B_1_ (AFB_1_) and cumoyl hydroperoxide (CHP) respectively. Tests were done in the presence and absence of metabolic activation S9 from rat liver [35, 37, 38]. Metabolic activation was achieved by an Aroclor 1254-induced S9 homogenate (0.7 nmol cytochrome P450 / mg protein) prepared from male Fischer rats [38] and incorporated in the S9 mixture at a level of 2 mg protein / ml.

### Mutagenicity testing

The ethyl acetate and aqueous methanol extracts were first tested for cytotoxicity using the standard plate incorporation method in the presence and absence of metabolic activation with S9 [39]. The assay consisted of the addition of an overnight bacterial culture (0.1 ml), the extract (0.1 ml) and/or pure compounds (0.1 ml)) and S9 mix (0.5 ml) to 2 ml of top agar at 45 °C. In the absence of S9 the latter was omitted. The mixture was vortexed, poured onto a minimal glucose agar plate and incubated at 37 °C for 48 h in the dark. Negative controls were included in the absence of the diagnostic mutagens, with DMSO as solvent in the presence and absence of the S9 mixture. DMSO was included in the controls as it was used to solubilize the extracts and in the preparation of the different dilutions and reported to be suitable for use in the *Salmonella* mutagenicity assay [40]. Mutagenicity testing was conducted at concentrations 10 times below cytotoxic doses. Five replicates were used for each concentration and all experiments were performed at least twice.

### Antimutagenicity testing

The antimutagenicity assay was conducted with the respective diagnostic mutagens for each strain in the presence of different dilutions of the extracts or pure compounds. Five replicates were used for each concentration and experiments were repeated at least once. The percentage inhibition was calculated as described previously [41].

### Total polyphenolic content and antioxidant activity

The total polyphenolic (TP) content of all extracts was done following the Folin-Ciocalteau (F-C) method by [42] with modifications [43, 44]. Five hundred microliter of EA and MeOH solution extracts were mixed with the F-C reagent. After 5 min 2 mL of Na_2_ CO_3_ solution (75 g/ L) was added, after 120 min standing in dark, the optical density was measured at 760 nm against a blank. The TP was calculated on the basis of the calibration curve of gallic acid and expressed as gallic acid equivalents (GAE), in milligrams per gram of the sample.

### Antioxidant assays

#### Ferric reducing antioxidant power assay (FRAP)

The ferric reducing antioxidant power (FRAP) of the extracts and samples was determined according to the method of [45]. The FRAP reagent was prepared by adding 10 mL of 10 mM 2,4,6-Tri(2-pyridyl)-s-triazine or 2,4,6-tri(2-pyridyl)-1,3,5-triazine (TPTZ) in 40 mM HCl, 10 mL FeCl_3_ in distilled water and 100 mL of the acetate buffer prior to use. Extracts were diluted in acetate buffer (300 mM, pH 3.6). A standard curve was generated using a 5 mM stock of (±)-6-Hydroxy-2, 5, 7, 8-tetramethylchromane-2-carboxylic acid (Trolox) dissolved in ethanol. The FRAP reagent (180 μL) was added to 20 μL of the sample/standard and incubated at 37 °C for 4 min and the absorbance was measured at 592 nm. Concentration for each sample was measured in duplicate, expressed as μmol trolox equivalents (TE) per gram of extract.

### 2′-Azino-bis (3-ethylbenzo-thiazoline-6-sulfonic acid) Diammonium salt (ABTS) assay

The ABTS radical scavenging activity of the extracts and samples was determined according to the method of [46]. The 2,2′-Azino-bis(3-ethylbenzo-thiazoline-6-sulfonic acid) diammonium salt (ABTS) reagent, dissolved in deionised water to yield a 7 mM solution, was prepared 12-16 h before use. The ABTS solution was diluted to yield an absorbance between 0.68 and 0.72 before use. Trolox, 1 mM, stock solution was used to generate a standard curve. The ABTS reagent (180 uL) was added to 20 μL of sample and incubated at 30 °C for 4 min and the absorbance measured at 734 nm. The percentage inhibition of absorbance for the standards and samples was calculated by using the blank (un-inhibited) as 100% and radical scavenging expressed as trolox equivalents per gram of extract.

### Statistical analyses

All individual groups were independent and tested for normality using the Kolmogorov-Smirnof Test. Levene’s Test was used to determine whether the groups had equal variances. Significant group differences were determined by the F-test (equality of variances) or the Welch Test (inequality of variances), while the post-hoc Tukey Test to separate means.

The Student’s Paired-sample Test was used to test for group differences when there were only two groups, with the Pooled method for groups with equal variances or the Satterthwaite method for groups with unequal variances. *P* < 0.05 indicated significant group differences.

## Results

The effects of sutherlandia extracts and compounds in antimutagenic and mutagenic assays are summarized in Tables 1, 2, 3 and 4.

**Table 1:** Antimutagenicity of the sutherlandia extracts on the four *S. typhimurium* tester strains. Inhibition expressed as % inhibition ± STD

Extract	Conc./plate (%)	TA97a (-S9)*	TA98 (+S9)	TA100 (+S9)	TA102 (-S9)
MeOH	50	18.4 ± 1.6^c^	(−) 48.9 ± 12.7^c#^	(−) 16.2 ± 5.6^c#^	72.0 ± 15.1^c^
	25	38.1 ± 6.3^b^	17.0 ± 3.6^b^	68.1 ± 3.7^b^	97.2 ± 1.4^b^
	10	85.4 ± 3.7^a^	91.4 ± 1.6^a^	83.2 ± 5.8^a^	114.5 ± 3.1^a^
EA	20	26.0 ± 3.3^c^	41.8 ± 2.5^c^	57.2 ± 5.8^c^	98.6 ± 3.9^c^
	10	64.8 ± 4.9^b^	69.7 ± 2.8^b^	75.5 ± 4.2^b^	109.0 ± 4.7^b^
	5	90.2 ± 3.2^a^	91.8 ± 3.7^a^	95.6 ± 3.9^a^	127.3 ± 10.4^a^
Diagnostic mutagen		9-AA (0.02 μg/plate)	2-AAF (5 μg/plate)	AFB_1_ (40 μg/plate)	CHP (0.1 μg/plate)
Revertant counts		369 ± 5	256 ± 6	373 ± 5	997 ± 99

*For TA97a concentration is 0.5, 1 and 2.5% for both extracts. The means of % inhibition of the three dilutions were compared for both extracts to determine significant differences between them. Mean % inhibitions followed with different letters indicate significant difference between them at *P* ≤ 0.01. Means followed by the same letters indicated no significant difference at P ≤ 0.01^#^ pro-mutagenicity of the 10% solution of the aqueous methanol extract

*Abbreviations*: *Conc* concentration, *MeOH* aqueous methanol extract, *EA* ethylacetate extract, *9-AA* 9-aminoacradine, *2-AAF* 2-acetamidofluorene, *AFB*_*1*_ aflatoxin B_1_, *CHP* cumoyl hydroperoxide

**Table 2:** Mutagenicity testing of sutherlandia extracts against four *S. typhimurium* tester strains

Extract	*conc. (%)	TA97a		TA98		TA100		TA102	
		(-S9)	(+S9)	(-S9)	(+S9)	(-S9)	(+S9)	(-S9)	(+S9)
MeOH	10	214 ± 3	237 ± 4	63 ± 3	69 ± 1	171 ± 3	184 ± 4	314 ± 61	387 ± 47
	50	191 ± 5	203 ± 3	77 ± 3	92 ± 5	117 ± 4	132 ± 5	264 ± 48	365 ± 9
EA	5	216 ± 3	271 ± 7	45 ± 3	53 ± 3	204 ± 3	221 ± 4	209 ± 21	297 ± 22
	20	191 ± 5	202 ± 2	63 ± 1	69 ± 1	174 ± 3	184 ± 3	210 ± 34	288 ± 25
Spontaneous revertants		185 ± 3	138 ± 5	31 ± 2	44 ± 3	102 ± 4	119 ± 2	185 ± 32	216 ± 41
Common Valid Range [39]		75-200	100-200	20-50	20-50	75-200	75-200	100-300	200-400
Revertant counts (Positive^#^control)		369 ± 5 (9-AA)		256 ± 6 (2-AAF)		373 ± 5 (AFB_1_)		997 ± 99 (CHP)	

Activity expressed as mean revertant counts ± STD (*n* = 5). * For TA97a concentration was 0.5 and 2.5% for both extracts. ^#^See details in Table 1 above. Where revertant counts are more than three times that of spontaneous revertant count, the compound is considered to be mutagenic [39]

*Abbreviations*: *9-AA* 9-aminoacradine, *2-AAF* 2-acetamidofluorene, *AFB*_*1*_ aflatoxin B_1_, *CHP* cumoyl hydroperoxide

**Table 3:** Antimutagenicity testing of the pure compounds against the four *S. typhimurium* tester strains

Compound	*conc. (%)	TA97a (-S9)	TA98 (+S9)	TA100 (+S9)	TA102 (-S9)
L-arginine	5	43.0 ± 0.9^a^	22.1 ± 8.4^a^	48.6 ± 2.5^a^	106.5 ± 1.0^a^
	2.5	57.1 ± 2.0^b^	67.4 ± 3.7^b^	96.0 ± 1.1^b^	116.1 ± 0.7^a^
	1	84.1 ± 1.8^b^	85.3 ± 3.6^b^	124.1 ± 2.4^c^	119.3 ± 0.6^a^
L-canavanine	5	(−) 101.9 ± 3.7^a^	25.1 ± 6.7^a^	38.9 ± 1.5^a^	101.1 ± 0.7^a^
	2.5	(−) 120.6 ± 4.5^a^	64.8 ± 5.0^b^	56.7 ± 2.1^b^	116.2 ± 0.4^a^
	1	(−) 138.2 ± 4.1^a^	80.1 ± 2.3^b^	92.2 ± 1.7^c^	125.9 ± 1.0^a^
D-pinitol	5	31.2 ± 8.8^a^	26.6 ± 7.3^a^	36.1 ± 1.8^a^	82.8 ± 3.5^a^
	2.5	79.0 ± 6.8^b^	48.8 ± 6.5^b^	79.6 ± 1.8^b^	109.5 ± 4.6^a^
	1	143.5 ± 16.6^c^	65.0 ± 10.3^b^	117.2 ± 3.0^b^	115.0 ± 5.3^a^
GABA	5	58.8 ± 3.4^a^	14.3 ± 7.9^a^	32.9 ± 2.1^a^	98.3 ± 1.0^a^
	2.5	61.8 ± 2.5^a^	45.8 ± 5.1^b^	66.2 ± 2.5^b^	113.1 ± 0.9^a^
	1	61.4 ± 1.4^a^	72.5 ± 3.0^b^	105.4 ± 1.5^c^	116.7 ± 0.4^a^
Positive control	Revertant counts	9-AA	2-AAF	AFB_1_	CHP
		247 ± 3	358 ± 26	360 ± 3	681 ± 4

Inhibition expressed as mean % inhibition ± STD of the positive control. All three mean % inhibitions of the pure compounds where compare at similar dilutions. The mM concentrations of the pure compounds (5%) were as follows: L-arginine [0.28 × 10^3^ mM], L-canavanine [0.26x10^3^mM], D-pinitol [0.25 × 10^3^ mM] and GABA (gamma aminobutyric acid) [0.48x10^3^mM]. *Note that GABA concentration is almost double that of the other three compounds. *This table also shows a dose-response increase of the antimutagenic activity of the compounds, where in most cases the highest concentration’s inhibition is more than double that of the lowest. The negative or minus (−) sign indicated co-mutagenicity of L-canavanine with 9-AA in the absence of S9 activation

*Abbreviations*: *9-AA* 9-aminoacradine, *2-AAF* 2-acetamidofluorene, *AFB*_*1*_ aflatoxin B_1_, *CHP* cumoyl hydroperoxide

**Table 4:** Mutagenicity testing of the pure compounds against the four *S. typhimurium* tester strains

Compound	*conc. (%)	TA97a		TA98		TA100		TA102	
		(-S9)	(+S9)	(-S9)	(+S9)	(-S9)	(+S9)	(-S9)	(+S9)
L-arginine	1	214 ± 3	204 ± 2	64 ± 3	68 ± 1	85 ± 3	87 ± 2	255 ± 3	263 ± 1
	2.5	167 ± 2	146 ± 2	64 ± 3	66 ± 3	72 ± 1	73 ± 4	236 ± 3	226 ± 2
	5	125 ± 2	113 ± 2	65 ± 4	60 ± 2	62 ± 2	68 ± 1	217 ± 3	206 ± 4
Spon. Revertants		223 ± 2	105 ± 3	37 ± 3	44 ± 3	105 ± 4	105 ± 2	293 ± 5	365 ± 3
L-canavanine	1	184 ± 3	187 ± 4	36 ± 2	102 ± 3	216 ± 4	284 ± 4	189 ± 5	294 ± 3
	2.5	155 ± 4	158 ± 3	34 ± 3	81 ± 3	182 ± 3	222 ± 3	168 ± 4	254 ± 3
	5	130 ± 4	145 ± 3	34 ± 3	59 ± 2	120 ± 3	201 ± 3	154 ± 4	189 ± 2
Spon. Revertants		196 ± 3	215 ± 4	51 ± 3	28 ± 2	204 ± 4	204 ± 4	270 ± 6	327 ± 4
D-pinitol	1	245 ± 4	200 ± 1	41 ± 3	97 ± 3	289 ± 5	312 ± 2	414 ± 2	460 ± 5
	2.5	205 ± 3	173 ± 2	42 ± 4	72 ± 2	275 ± 6	288 ± 6	395 ± 3	465 ± 3
	5	186 ± 4	158 ± 5	42 ± 2	54 ± 1	241 ± 4	267 ± 4	386 ± 4	467 ± 1
Spon. Revertants		173 ± 3	184 ± 3	42 ± 3	50 ± 1	204 ± 2	204 ± 2	378 ± 2	526 ± 4
GABA	1	230 ± 4	152 ± 8	24 ± 1	117 ± 3	233 ± 4	286 ± 3	188 ± 3	385 ± 4
	2.5	175 ± 4	134 ± 3	25 ± 3	87 ± 2	195 ± 4	288 ± 7	175 ± 3	318 ± 4
	5	128 ± 6	138 ± 6	24 ± 1	61 ± 3	141 ± 2	288 ± 3	162 ± 3	290 ± 2
Spon. Revertants		196 ± 3	215 ± 4	28 ± 2	51 ± 3	204 ± 4	204 ± 4	270 ± 6	327 ± 4
Common Valid Range [39]		75-200	100-200	20-50	20-50	75-200	75-200	100-300	200-400

Activity expressed as mean revertant counts ± STD. *Where revertant counts are more than three times that of spontaneous revertants, the compound is considered to be mutagenic [39]

### Dose response effects

Dose response effects were observed in the following two ways: Typical dose response where the inhibitory effect was directly related to the concentration of the extracts and pure compounds against 2-AAF, AFB_1_, CHP and 9-AA (Tables 1 and 3); and a saturation effect where there was constant inhibition across the three concentrations tested with all pure compounds against CHP and GABA against 9-AA (Table 3).

### Sutherlandia-mutagen interactions

The sutherlandia extract-mutagen interactions were observed to be either mutagenic / co-mutagenic or antimutagenic.

### Mutagenicity/co-mutagenicity

The sutherlandia mutagenicity was observed in the following two ways:

(i) Pro-mutagenic activity of the MeOH extract (50%) in the presence of S9, against 2-AAF and AFB_1_ – this was exhibited by the negative percentage found, implying that the number of revertants was higher than that of the positive control (Table 1). However, none of the extracts exhibited a positive mutagenic response when compared to the spontaneous background counts of each strain (Table 2). At least a three-fold increase in the background count was taken as a mutagenic response as reported elsewhere [39]
(ii) Co-mutagenic activity by L-canavanine not requiring S9 against 9-AA in a dose response manner (Table 3). None of the sutherlandia extracts nor the pure compounds elicited a mutagenic response when considering a 3-fold increase above the spontaneous revertant counts of the different *Salmonella* strains (Table 4).

### Antimutagenicity

Antimutagenicty was seen in the decrease in the number of revertants relative to the positive control implying a protective effect (Table 1). Thus the EA and MeOH extracts exhibited a dose dependent antimutagenic effect against direct and indirect acting mutagens. The EA extract showed a significantly higher antimutagenic potency against both these mutagens, and a significantly high activity against all four strains at the 10% concentration which was the only common dilution level between the two extracts (Table 1). The 10% concentration of the MeOH extract on the other hand exhibited a pro-mutagenicity in the presence of the S9 in TA98 with 2-AAF and TA100 with AFB_1_ as mentioned above (Table 1).

The four pure compounds were tested at concentrations of 5% and lower as higher concentrations exhibited cytotoxicity (Table 3). Overall, all four pure compounds showed varying levels of inhibition against the mutagenicity of the mutagens, either in the presence or absence of S9 at three dilutions. However, L-canavanine showed a co-mutagenic response only with 9-AA in the absence of S9. A typical dose response manner of inhibition were obtained which significantly differed between the three dilutions for most of the compounds.

### The total polyphenol content and antioxidant activity

The MeOH extract had 4.19 μg GE / mg total phenolic content and EA had 12.56 μg GE / mg, a three-fold higher level. The EA extract also showed a higher antioxidant activity with both assays, than the MeOH extract. The FRAP and ABTS assays showed that the EA extract had a higher antioxidant activity than the MeOH (results not shown).

## Discussion

Gomes-Carneiro et al. [47] demonstrated that in the Salmonella/microsome assay, antimutagenicity generally manifests as a reduction in the number of revertant colonies caused by a known genotoxic agent, and cytotoxicity to tester strains may also result in a reduction of revertants. The concentrations of the treatments were optimized to be in the non-toxic range. The tester strains used are commonly used to screen mutagenicity resulting from frame shift (TA97a and TA98), base pair (TA100) and oxidate and cross-linking (TA102) [48]. The spontaneous revertants were found to be within the common valid range for the strains [38]. The reason why some strains were tested only in the absence of S9 is because they do not need enzymatic metabolic activation to exhibit their mutagenicity.

In this study mutagens with different chemical structures and mechanisms of action were used to determine the protective effect of extracts of *Sutherlandia frutescens*. Both extracts exhibited a protective effect, with the EA extract exhibiting a greater potency than the MeOH extract. Pro-mutagenic activity of the latter was observed, in the presence of S9 against 2-AAF and AFB_1_ at the highest concentration level (50%). At the 25 and 10% concentrations however, the MeOH extract exhibited an antimutagenic response against both mutagens. An apparent stabilization of the S9 in the mutagenicity test could have contributed to the enhanced metabolic activation recorded at the higher concentration of the extract. This biphasic mode of action has been reported in the past for many natural compounds /extracts including flavonoids [49] which have been called the “Janus carcinogens and mutagens”, which is concentration dependent [50–52]. An interesting response was noticed with L-canavanine which, in the absence of metabolic activation exhibited a co-mutagenic effect with 9-AA in a dose dependent manner. Although the mechanism of this co-mutagenic effect is not know it seems to support the view that L-canavanine may exhibit carcinogenic properties [53, 54].

EA extracts showed superior antimutagenic activity in all the tester strains and can also be deemed to be more potent as activity was at lower concentrations compared to MeOH. The higher antimutagenic effect and higher antioxidant activity of the EA extract can be attributed to the higher total phenolic content. Total polyphenol content correlated with superior antioxidant activity as would be expected while it also seems to be associated with the increased anti-mutagenic effects in this case. The co-mutagenicity exhibited by L-canavanine with 9-AA in the absence of S9 and the pro-mutagenic response of the highest concentration of the MeOH extract with 2-AAF and AFB_1_ in the presence of S9 is of interest. It is not clear whether any of the known compounds tested, specifically L-canavanine, did play a role in the pro-mutagenic effects of the MeOH extract. This is based on the fact that L-canavanine is co-mutagenic against TA97a in the absence of S9 but the crude MeOH extract (50%) is pro-mutagenic against both TA98 and TA100 (in the presence of S9). This could be due to the fact that L-canavanine is less co-mutagenic in combination with the other amino acids and/or that other compounds in the MeOH extract are responsible.

Edenharder et al. [50] have demonstrated the importance of structure-activity relationships (SARs) in antimutagenic activity. From their study they demonstrated that flavones and flavonols were the most active and their activity increased with the number and position of hydroxyl functions, while isoflavones and flavanones and their glycosides, were inactive. SARs are also important in antioxidant activity [55, 56]. Free radical scavenging and induction of antioxidant enzymes have been shown to be responsible for antimutagenic activity [57]. In this study the EA extract which showed superior antioxidant activity also demonstrated better antimutagenic activity. Antimutagenic activity can be affected by a variety of factors such as chemical and/or enzymatic inactivation, scavenging of reactive oxygen species (ROS) or prevention of the formation of mutagenic metabolites [52].

Elgorashi et al. [4, 35] have previously reported on the mutagenicity and antimutagenicity of South African medicinal plants including sutherlandia. Our results concur with those of [35] who showed antimutagenic activity in DCM (a non-polar extract) and 90% (*w*/w) methanol crude extracts of sutherlandia. They obtained much higher inhibitions against TA98 and TA100 in contrast to lower inhibitions with the non-polar EA extract in this study. This study was more comprehensive in that it tested two extracts and four compounds against all four commonly used strains. EA showed a higher activity against TA98 and TA100 respectively in the presence of S9 than in the previous study by [35]. The S9 homogenate appears to metabolize compounds in EA extract thus enhancing the antimutagenic activity. Conversely, these drug metabolizing enzymes appear to promote the pro-mutagenicity of the aqueous methanol (50%) extract. The antimutagenicity at 25 and 10% dilutions of the aqueous methanol extracts could be related to biphasic dose response effects as mentioned above and previously reported [58]. However, further investigation as, apart from the dose, on the differential effects of the metabolizing enzymes on different compounds should also be of interest. These equivocal actions have been previously reported with the antimutagenic properties of rooibos extracts [59].

The activity of the four compounds tested has not been reported previously. A typical dose response indicating an apparent saturation effect was observed with all the compounds. L-canavanine showed co-mutagenic rather than pro-mutagenic effects. A pro-mutagen is a synergistic agent that activates mutagenicity at low concentrations and later becomes antimutagenic at higher concentrations [50, 52]. A co-mutagen, on the other hand enhances mutagenicity without enzymatic activation [60]. Rosenthal [61] reported that L-canavanine has anticancer activity. The pro-mutagenicity shown in the presence of metabolic activation with 2_AAF against TA 97a by the 10% solution of the MeOH extract is likely not attributed to L-canavanine when considering the co-mutagenicity observed against TA97a with 9-AA in the absence of metabolic activation. As the current investigation did not include L-canaline, it is not clear whether this major metabolite, of L-canavanine exhibites pro- or co- mutagenicity.

No amino acids were found/and or observed in the EA extract, as confirmed by the TLC which we developed with ninhydrin spray reagent (results not shown). These compounds being amino acids were observed (through TLC) to be present in the 50% aq MeOH extract. L-canavanine, L-arginine, GABA and D-pinitol are extractable with more polar solvents (water and/or methanol or a mixture of both). This was confirmed by TLC. The absence of L-canavanine may explain in part the absence of pro-mutagenicity and/or co-mutagenicity in the EA fraction. The antimutagenic activity exhibited by the EA extract is therefore not due to the amino acids tested (as these were absent in this extract), but may be due to the presence of other compounds such as the less polar sutherlandosides and phenolic glycosides. Hatami et al. [44] showed that polar extracts contained higher phenolic content as well as higher antioxidant activity, which is in contrast to the present study where the EA extract showed a higher phenolic content and a higher antioxidant activity. These phenolic are most likely in glycosylated form which is why they more easily partition into EA.

Plants play a major role in chemoprevention, which is recognized as a plausible and cost-effective approach to reduce cancer morbidity and mortality by inhibiting precancerous events before induction/activation of the clinical stages [61, 62]. In this regard it is important to test for both mutagenic and anti-mutagenic potential of medicinal plant extracts. The absence of a mutagenic response by plant extracts against *Salmonella* strains, in the Ames assay is a positive step towards determining their safety [35]. However, plant extracts exhibiting a mutagenic effect need to be further investigated to determine possible genotoxicity [35]. Those with mutagenic potential should be used with caution if at all. On the other hand, plant species exhibiting antimutagenic activity may play a role in chemoprevention.

The Ames assay was used in this study as it is reliable, quick and easy. This test is used to screen for possible carcinogens and mutagens. However, if a substance is screened and does not give a mutagenic response, it only suggests that the substance is not mutagenic to the particular bacterial strain it is tested against and for the genetic endpoint tested [35]. Generally, when testing for carcinogenicity and antimutagenicity, a follow-up in vivo test would have to be performed in mice and/or rats of both sexes [51, 63].

## Conclusion

The current study is the first report on the mutagenicity and antimutagenicity of *S. frutescens* crude extracts and comparing its polar and non-polar extracts as well as pure compounds. This study has at least in part, explained the pharmacological potential of non-protein amino acids from sutherlandia and that of organic and aqueous extracts. Only L-canavanine showed a co-mutagenic effect with the indirect mutagen 9-AA but it is not clear whether it is responsible for the pro-mutagenic activity of the MeOH extract with 2-AAF and AFB_1_ in the presence of S9. The practical implications are that formulations of aqueous sutherlandia preparations should contain little or no L-canavanine because of the potential for cellular genotoxicity shown here while formulations using less polar solvents may have a better risk profile.

## Abbreviations

2-AAF: 2-acetamidofluorene
50% MeOH: 50% aqueous methanol
9-AA: 9-aminoacridine
ABTS: 2′-Azino-bis (3-ethylbenzo-thiazoline-6-sulfonic acid) Diammonium salt
AFB_1_: Aflotoxin B_1_
CHP: Cumoyl hydroperoxide
DMSO: Dimethyl Sulphoxide
EA: Ethylacetae
F-C: Folin-Ciolcalteau
FRAP: Ferric reducing antioxidant power
G-6-P: Glucose-6-phosphate
GABA: γ-amino butyric acid
GAE: Gallic acid equivalents
MeOH: Methanol
NADP: Nicotinamide adenosine diphosphate
SF: *Sutherlandia frutescens*
TE: Trolox equivalents
TLC: Thin layer chromatography
TP: Total phenolic content
TPTZ: 2,4,6-Tri(2-pyridyl)-s-triazine or [2,4,6-tri(2-pyridyl)-1,3,5-triazine]
